# Impact of water fluoridation on dental caries decline across racial and income subgroups of Brazilian adolescents

**DOI:** 10.4178/epih.e2022007

**Published:** 2022-01-03

**Authors:** Rafael Aiello Bomfim, Paulo Frazão

**Affiliations:** 1 Department of Community Health, Federal University of Mato Grosso do Sul (UFMS), Campo Grande, Brazil; 2 Public Health School, University of São Paulo, São Paulo, Brazil

**Keywords:** Water fluoridation, Oral health, Dental caries, Ethnicity, Adolescent health

## Abstract

**OBJECTIVES:**

The aim of this study was to assess the impact of community water fluoridation (CWF) on differences in dental caries decline across racial and socioeconomic subgroups of Brazilian adolescents.

**METHODS:**

Two nationwide Brazilian population-based oral health surveys were used (Brazilian Oral Health Survey 2003 and 2010). In total, 7,198 adolescents from 15 years to 19 years old living in 50 cities investigated in both surveys were included. The mean numbers of untreated decayed teeth (DT) according to racial (Whites vs. Browns/Blacks) and socioeconomic subgroups (at or above the minimum wage per capita vs. under) were analysed. Difference-in-differences negative binomial regressions were adjusted by schooling, age, and sex. Decayed, missing, and filled teeth and DT prevalence, calculated as a categorical variable, were used in sensitivity analyses.

**RESULTS:**

The adjusted difference of reduction in DT was similar across socioeconomic subgroups (β=-0.05; 95% confidence interval [CI], -0.45 to 0.35) and favoured, but not to a significant degree, Whites (β=-0.34; 95% CI, -0.74 to 0.04) compared to Brown/Blacks in fluoridated areas. In non-fluoridated areas, significant differences were observed in the mean number of DT, favouring the higher socioeconomic subgroup (β=-0.26; 95% CI, -0.53 to -0.01) and Whites (β=-0.40; 95% CI, -0.69 to -0.11) in relation to their counterparts. The sensitivity analyses confirmed the findings.

**CONCLUSIONS:**

The similar reduction in DT across income subgroups suggests that CWF has had a beneficial effect on tackling income inequalities in dental caries within a 7-year timeframe.

## INTRODUCTION

Dental caries is one of the most prevalent diseases globally. Untreated dental caries affect 35% of the worldwide population (2.4 billion people), with a disproportionate impact on socioeconomically disadvantaged and vulnerable groups [1].

The preventive effects of conjoint exposure (e.g., use of fluoride toothpaste in a fluoridated area) are additive and lower than the sum of each one’s effect [2]. Moreover, community water fluoridation (CWF) is associated with reduced dental ambulatory sensitive hospitalizations [3], and its cessation was found to increase the mean number of decayed primary teeth [4].

Although information on the impact of water fluoridation on inequalities in dental caries remains insufficient [5], some studies have shown that CWF has the potential to reduce race-related and income-related inequalities [6-8]. For example, in Brazil, which has the world’s largest population of African descent outside of the native continent, a study showed that CWF was associated with reduced racial inequalities in dental caries in deprived settings [9].

The use of fluoride is a breakthrough in public health, and resolution 6,017 from the World Health Assembly confirmed the relevance of CWF policy for promoting oral health [10]. More than a political issue, CWF is an ethical issue [11]. Communities that have ceased fluoridation showed increased values of dental caries in children [4,12,13]. Australian children from 9 years to 14 years old with 100% lifetime exposure to fluoridated water had significantly fewer dental caries than those with lower exposure [14]. Comparing the United States counties with distinct levels of water fluoridation coverage (≥ 75 vs. < 75%), researchers found a difference of 12% in dental caries experience in children and adolescents aged 6-17 years old [15].

A randomized controlled trial is a study design essential for assessing clinical interventions; however, it is challenging to deploy to evaluate the efficacy of population-based public policies such as water fluoridation. Therefore, other methods specifically designed for causal inference in observational data have been proposed for impact assessment [16,17]. In Brazil, the fluoridation of public water supplies has been mandatory since 1974, according to Federal Law 6050. However, it is estimated that 40% of Brazilian cities are not supplied CWF through this beneficial public health policy [18]. Nonetheless, the prevalence and severity of dental caries has been declining in Brazil, while income inequalities persist [19]. To the authors’ knowledge, no study has compared differences in the decline of dental caries across income and racial subgroups between 2 time points in fluoridated and non-fluoridated areas utilising a difference-in-differences (DiD) approach [17]. Although this design permits the investigation of a potential association between policy implementation and the measured outcome, the main focus was to verify whether the dental caries decline differed across racial and income subgroups.

The aim of this study was to assess the impact of CWF on differences in the dental caries decline across racial and socioeconomic subgroups of Brazilian adolescents between 2003 and 2010 in the context of widespread use of fluoride toothpaste.

## MATERIALS AND METHODS

Data came from the latest 2 nationwide population-based oral health surveys, which were conducted in 2003 [20] and 2010 [21] (Brazilian Oral Health Survey [SBBrazil-2003 and 2010]). In 2003, the survey was conducted in 250 municipalities, while it was carried out in 177 municipalities in 2010. Probability cluster sampling was used to select children, adolescents, adults, and elders. Only individuals from 15 years to 19 years old living in cities investigated in both surveys were included in the present analysis.

Both surveys’ interviews and clinical examinations followed the criteria recommended by the World Health Organisation (WHO) [22] for measuring dental caries, and were carried out in respondents’ homes by teams consisting of a general dentist and an assistant. Handbooks were developed to guide the procedures of sampling and data collection in each survey. Depending on the field characteristics, 2-5 dental examiners were selected and underwent between 24 hours and 32 hours of training and calibration in each municipality alone or in groups of 2 municipalities or 3 municipalities. Kappa values above 0.65 were considered acceptable in both surveys [20,21]. Only examiners with kappa values above 0.65 were approved for data collection.

The outcome was the mean number of untreated decayed teeth (DT), defined as a count variable that estimated each participant’s current disease severity. According to the WHO criteria, DT was defined as a tooth with a lesion in the pit and fissure; a smooth tooth surface with an unmistakable cavity, undermined enamel, or a detectably softened floor or wall; or a temporary restoration (except glass ionomer) [22]. A Community Periodontal Index probe was used to confirm visual evidence of caries on the occlusal, buccal, and lingual surfaces. We considered the tooth, and not the surface, as the unit parameter.

The investigated intervention was the provision of CWF. Fifty Brazilian cities were investigated in both the 2003 and 2010 oral health surveys, and 25 of them had provided CWF since at least 2000. They were grouped into non-fluoridated and fluoridated areas. Information on CWF was obtained from 2 different data sources using information provided by water companies and municipal water surveillance [18,23,24].

The effect of CWF was assessed according to different categories of racial and income subpopulations. The racial variable was based on the respondent’s self-assessment considering categories used by the Brazilian Institute for Geography and Statistics, based on skin colour. The categories included Whites, Asians, Browns (mixed ethnicity), Blacks, and Indigenous groups [25]. As proportions of adolescents belonging to Indigenous (0.8%) and Asian (1.0%) groups were very small in both independent samples, they were not included in the final analysis. Income was measured using equivalised per capita monthly household income and dichotomised to distinguish between families living below the Brazilian minimum wage (< 1 MW) and those at or above MW (≥ 1 MW) [26]. In December 2003, the monthly Brazilian MW was Brazilian real (BRL) 240 or US dollar (USD) 83.33. In December 2010, the Brazilian MW was BRL 510.00 or USD 301.70. The covariates were age in years, sex (male or female), and schooling (< 4 or ≥ 4 years).

The descriptive analyses included cross-tabulations of the outcome for racial (Whites vs. Browns/Blacks) and equalised income (< 1 or ≥ 1 MW) subgroups according to the CWF. Point estimates and 95% confidence intervals (CIs) were used to interpret the mean outcome values. Both areas experienced declining trends in dental caries [17]. There was no change in the drinking water status during the study period. As the focus was to investigate reduction differences across racial and income subgroups, the fact that the CWF intervention began at least 3 years before the baseline data did not undermine the analysis undertaken herein.

We regressed DiD stratified by CWF exposure. In the stratum of fluoridated areas, the association between the outcome and categories of racial subgroups (Browns/Blacks vs. Whites) and socioeconomic subgroups (lower vs. upper income), with an interaction term (i.e., the year) was investigated. The same was done in the stratum of non-fluoridated areas (control units). This analysis quantified the difference in the reduction of the outcome between the years across racial and socioeconomic subgroups. Due to the overdispersion of the outcome values, coefficients across racial and socioeconomic subgroups were estimated using negative binomial regression. All analyses were adjusted by sex, age, and schooling. The beta-coefficient (β) was interpreted as the mean reduction of a group compared to its reference group with a 95% CI. Moreover, the percentage difference values resulting from the quotient between the β-coefficient and the baseline mean of the reference group were also reported [27].

Analyses were carried out using Stata version 14.2 (StataCorp., College Station, TX, USA). The sampling weights available in the database were employed. All analyses were based on complete cases (without missing values) in the independent samples. A comparison of the total sample with the analysed samples showed no significant differences in any outcome or covariates (Supplementary Material 1). Sensitivity analysis stratifying the number of DTs as dichotomous (0: without caries and 1: ≥ 1 DT) (Supplementary Material 2) and using decayed, missing, and filled teeth were done (Supplementary Materials 3-5). We used the STROBE (Strengthening the Reporting of Observational Studies in Epidemiology) guidelines to report the results of the present investigation.

### Ethics statement

This investigation complies with the guidelines for human studies and includes evidence that the research was conducted ethically following the World Medical Association Declaration of Helsinki. All subjects provided written informed consent, and the institute’s committee for human research approved the study protocol. Ethical approval for the SBBrazil 2010 was granted by the Ethics Health Commission (CNS) (resolution 15498) on July 1, 2010, and permission for the SBBrazil 2003 survey was granted on July 21, 2000 (resolution CNS 1.356, process 25.000.009.632/00-51).

## RESULTS

In total, 7,198 adolescents comprised the analytical sample. Table 1 shows the distribution of the sample’s characteristics as a whole. In both 2003 and 2010, the 95% CIs of the mean numbers of untreated DT were significantly higher in the non-fluoridated than fluoridated areas, and the mean number of DT was higher in the lower-income group. In addition, the mean number of DT was significantly higher in Browns/Blacks than in Whites in 2010.

Adolescents self-declared as Browns/Blacks had higher numbers of DT than their counterparts in non-fluoridated areas in 2010. Those living in families with an income under 1 MW had higher numbers of untreated dental caries in non-fluoridated areas in both years and in fluoridated areas in 2010 (Table 2).

Table 3 shows the crude and adjusted DiD analysis outputs as mean numbers of DT across income and racial subgroups according to exposure to CWF from 2003 to 2010. The crude and adjusted outputs showed significant differences only in non-fluoridated areas. Adolescents living in higher-income families and those self-declared as Whites had significantly higher reductions in the mean number of DT (12.4 and 14.8%, respectively) than their counterparts. Contrarily, the differences were non-significant in fluoridated areas, although more favourable trends were found for Whites (β= -0.34; 95% CI, -0.74 to 0.04) than in Blacks/Browns. Supplementary Material 1 presents an analysis of the missing values. A sensitivity analysis based on the percentages of individuals with at least 1 untreated DT confirmed our findings (Supplementary Material 2). In fluoridated areas, the reduction in the proportion of people with at least 1 DT was similar across racial and socioeconomic groups, while in non-fluoridated areas, the reduction was more prominent for adolescents from wealthier families and Whites. Supplementary Materials 3-5 showed higher values of DMFT in non-fluoridated areas, confirming our findings.

## DISCUSSION

No difference was found in the reduction of DT mean values between income subgroups in response to CWF, suggesting favourable trends in health equity amongst Brazilian adolescents; instead, in non-fluoridated areas, the trends favoured adolescents from wealthier families and Whites.

In both areas, we observed a reduction in the mean number of DT. This declining trend has been observed worldwide due to the widespread use of fluoride [2]. Investigations showed that dental caries have also been decreasing in frequency in the overall young Brazilian population, but these improvements have not been similar among socioeconomic groups [19,28]. However, those studies did not compare changes according to exposure to CWF. Our findings showed similar decreases across socioeconomic subgroups in the mean values of DT in fluoridated areas, while the differences remained in non-fluoridated areas. In a study of Australian children, all inequality indices indicated that caries experience was concentrated among lower-income groups and was lower in fluoridated than in non-fluoridated areas [29].

Regarding racial subgroups, although the differences in DT reduction were non-significant, they were as wide as those observed for income subgroups. Considering the rates of individuals with at least 1 untreated DT (Supplementary Material 3), the differences in reduction were compatible, with similar values for both income and racial subgroups in fluoridated areas but higher reductions in wealthier groups and Whites in non-fluoridated areas. As the income differences were estimated with adjustment for racial groups and vice versa, this finding could mean that income differences are more amenable to being addressed by CWF than racial differences. This hypothesis should be confirmed in further DiD analyses. Previous evidence has been derived from cross-sectional studies [30]. One showed that CWF did not reduce racial inequalities significantly [31], while the other observed no significant ethnic inequalities in deprived settings with CWF [9].

Race has emerged as a factor explaining the persistence of health inequalities in some contexts [32]. The relative disadvantage that racial groups face in terms of oral health has been interpreted as stemming from structural or macro-level processes. This includes dental care, living in deprived neighbourhoods, and restricted access to dental care and fluoridated piped water [33]. These inequalities persisted in models adjusted for sex, schooling, income, and age. In general, those from higher socioeconomic status (SES) are aware of the risks of caries and have the resources—money, knowledge, power, prestige, and beneficial social connections—to engage in prevention rather than treatment [34]. Our study demonstrated that even adjusting for schooling and income (an inherent part of SES), lower health gains for Browns/Blacks than for Whites were observed in non-fluoridated areas. In other words, this finding means that race and SES did not overlap. A possible explanation could be related to difficulties in accessing fluoridated toothpaste by different racial groups [35].

In non-fluoridated areas, the reduction of the mean number of untreated DT was higher in adolescents from wealthier families than in their counterparts. A decomposition analysis of dental caries experience among 9-14-year-old Australian children showed that exposure to CWF explained one-third of area-level income inequalities [36]. Other studies have shown the effect of CWF on reducing dental caries in the context of multiple sources of fluoride [5,8,14,15,37]. Spencer et al. [14] found that income-related inequality in caries was lower in fluoridated than in non-fluoridated areas for Indigenous and non-Indigenous children. One study showed benefits associated with higher lifetime exposure to CWF even adjusting for socioeconomic conditions, and another showed potential benefits of CWF on income inequalities [38], corroborating our findings.

Our study has some strengths and limitations. First, this DiD approach used data from 2 national epidemiological surveys that accurately reflected the country’s characteristics as a whole. We utilized data from the 50 cities that participated in the 2003 and 2010 surveys, so the cluster units (cities) were the same. Second, water fluoridation levels were determined using different data sources, thereby improving their reliability. Third, unlike other dental caries measurement tools, the selected outcome (mean number of untreated DT) measures the current disease severity, which is an indicator less affected by differences in access to oral healthcare and by the dentist’s decision to perform a restoration. The outcomes (mean number of untreated DT) were compared across socioeconomic and racial subgroups according to exposure to CWF in a 7-year period. They showed the impact of CWF independently of other dental caries determinants that affected both the exposure and non-exposure group [16]. A limitation is that data on exposure to fluoride at the individual level were not available. In the DiD approach, the baseline data are expected to be at the beginning of the intervention, whereas in the current study design, we are sure that the intervention began at least 3 years before, according to available official data from national sanitation research gathered in 2000. As the investigated intervention was measured in a defined age group that was exposed and non-exposed to CWF during the group’s lifetime, the difference between those times does not undermine the analysis. The ideal scenario would be that all cities included in the fluoridated area had begun the public policy implementation simultaneously and at least 10 years before, so that effects could be observed in the permanent dentition of 15-19-year-old adolescents. As this paper is the first to report the effects of CWF through a DiD analysis, further studies are needed to elucidate points not addressed herein. The complexity involved in racial classification in Brazil must also be highlighted [9,39]; however, this study used the best data representing the Brazilian population of adolescents as a whole.

In conclusion, the similar reduction in DT across income subgroups suggests that CWF had beneficial effects on tackling income inequalities in dental caries within a 7-year timeframe.

## Figures and Tables

**Table 1.** Descriptive characteristics and weighted mean numbers of DT in Brazilian adolescents

Characteristics	Total (n=7,198)	% (95% CI)	DT
			Mean (95% CI)
Racial group 2003 (n=3,178)			
Whites	1,254	41.5 (36.7, 46.7)	1.94 (1.50, 2.37)
Browns and Blacks	1,924	58.5 (53.6, 63.3)	2.23 (1.96, 2.50)
Racial group 2010 (n=4,020)			
Whites	1,653	43.0 (40.5, 45.6)	1.24 (1.04, 1.44)
Browns and Blacks	2,367	57.0 (54.4, 59.5)	2.04 (1.86, 2.23)
Per capita income 2003			
Under MW	1,641	48.6 (43.7, 53.4)	2.57 (2.19, 2.94)
At and above MW	1,537	51.4 (46.5, 56.3)	1.67 (1.41, 1.93)
Per capita income 2010			
Under MW	2,158	54.2 (51.6, 56.7)	2.08 (1.88, 2.29)
At and above MW	1,862	45.8 (43.3, 48.4)	1.24 (1.08, 1.41)
Fluoridation 2003			
No	1,596	27.5 (24.5, 30.6)	2.83(2.60, 3.07)
Yes	1,582	72.5 (69.4, 75.5)	1.83 (1.52, 2.14)
Fluoridation 2010			
No	1,770	26.7 (24.8, 28.8)	2.39 (2.17, 2.62)
Yes	2,250	73.3 (71.2, 75.2)	1.44 (1.28, 1.61)

DT, decayed teeth; CI, confidence interval; MW, per capita minimum wage.

**Table 2.** Weighted mean numbers of DT in Brazilian adolescents, according to fluoridation at the urban level (n=7,198)

Individual variables	n	DT	n	DT
		Non-fluoridated		Fluoridated
Ethnic group 2003	1,596		1,582	
Whites	422	2.47 (2.09, 2.85)	832	1.81 (1.29, 2.35)
Browns and Blacks	1,174	2.98 (2.69, 3.27)	750	1.85 (1.49, 2.21)
Ethnic group 2010	1,770		2,250	
Whites	573	1.51 (1.19, 1.84)	1,080	1.17 (0.96, 1.53)
Browns and Blacks	1,197	2.85 (2.57, 3.13)	1,170	1.68 (1.45, 1.93)
Per capita income (equivalised) 2003	1,596		1,582	
Under MW	937	3.11 (2.81, 3.41)	704	2.28 (1.73, 2.85)
At or above MW	659	2.41 (2.20, 2.80)	878	1.47 (1.17, 1.79)
Per capita income (equivalised) 2010	1,770		2,250	
Under MW	1,019	2.96 (2.64, 3.28)	1,139	1.75 (1.50, 2.01)
At or above MW	751	1.69 (1.41, 1.98)	1,111	1.09 (0.89, 1.28)

DT, decayed teeth; MW, per capita minimum wage.

**Table 3.** Diff-in-Diff analysis of the average number of DT amongst Brazilian adolescents stratified by exposure to CWF (n=7,198)

Variables			DT stratified by exposure to CWF						
			n	Mean 2003	n	Mean 2010	Diff-in-Diff^1^	Diff-in-Diff^2^	%D
Non-fluoridated areas									
	Socioeconomic groups								
		Under 1 MW	937	3.11	1,019	2.96	-0.30 (-0.57, -0.02)	-0.26 (-0.53, -0.01)	-12.45
		At or above 1 MW	659	2.41	751	1.69	1.00 (reference)	1.00 (reference)	
	Racial groups								
		Browns and Blacks	1,174	2.98	1,197	2.85	-0.44 (-0.74, -0.14)	-0.40 (-0.69, -0.11)	-14.77
		Whites	422	2.47	573	1.51	1.00 (reference)	1.00 (reference)	
Fluoridated areas									
	Socioeconomic groups								
		Under 1 MW	704	2.28	1,139	1.75	-0.04 (-0.43, 0.35)	-0,05 (-0.45, 0.35)	-2.72
		At or above 1 MW	878	1.47	1,111	1.09	1.00 (reference)	1.00 (reference)	
	Racial groups								
		Browns and Blacks	750	1.85	1,170	1.68	-0.34 (-0.77, 0.07)	-0.34 (-0.74, 0.04)	-18.38
		Whites	832	1.81	1,080	1.17	1.00 (reference)	1.00 (reference)	

Values are presented as β coefficient (95% confidence interval). Diff-in-Diff, difference-in-difference; CWF, community water fluoridation; %D, percentage of difference (β coefficient/baseline mean of the reference group); MW, per capita minimum wage; DT, decayed teeth.

1 Unadjusted.

2 Adjusted for schooling, age, sex, income (if analysing racial groups) and racial group (if analysing income).

## SUPPLEMENTARY MATERIALS

Supplementary Material 1. Comparision whole sample and complete cases weighted proportions and means in Brazilian adolescents

Supplementary Material 2. Difference in Differences analysis in untreated caries presence (0 and ≥1) in Brazilian adolescents ( n=7198)

Supplementary Material 3. The Difference-in-Differences analysis in average values of number of DMFT amongst Brazilian adolescents and stratified by exposure to CWF (n=7198)

Supplementary Material 4. Descriptive characteristics and weighted means of DMFT of Brazilian adolescents (n=7198)

Supplementary Material 5. DMFT weigthed means of Brazilian adolescents, according to Fluoridation contexts (n=7198)
