# Supplementary material for: Impact of water fluoridation on dental caries decline across racial and income subgroups of Brazilian adolescents
Source: Epidemiol Health. 2022 Jan 3;44:e2022007. doi: 10.4178/epih.e2022007 (PMC9016390; doi:10.4178/epih.e2022007)
Supplement: Supplementary Material 1. — Comparision whole sample and complete cases weighted proportions and means in Brazilian adolescents [file epih-44-e2022007-suppl1.docx]

**Supplementary Materials**

| SupplementaryMaterial 1. Comparision whole sample and complete cases weighted proportions and means in Brazilian adolescents | | | | | | |  |
| --- | --- | --- | --- | --- | --- | --- | --- |
| **Individual Variables (whole sample)** | | (n=7890) |  | **Complete Cases** |  | (n=7198) | **p-value** |
|  | n | % (CI 95%) |  |  | n | % (CI 95%) |  |
| **Ethnic Group 2003** | 3313 |  |  | **Ethnic Group 2003** | 3178 |  |  |
| Whites | 1288 | 40.7 (36.1-45.5) |  | Whites | 1254 | 41.5 (34.1-49.3) | 0.23 |
| Pardos | 1762 | 49.2 (44.4-53.9) |  | Brown | 1674 | 48.2 (40.2-56.3) |  |
| Blacks | 263 | 10.1 (7.9-12.9) |  | Blacks | 250 | 10.3 (06.1-16.9) |  |
| **Ethnic Group 2010** | 4358 |  |  | **Ethnic Group 2010** | 4020 |  |  |
| Whites | 1796 | 43.5 (41.0-45.9) |  | Whites | 1653 | 43.0 (36.4-49.9) | 0.33 |
| Pardos | 2058 | 43.4 (41.0-45.8) |  | Brown | 1897 | 43.5 (36.0-51.3) |  |
| Blacks | 504 | 13.1 (11.5-14.9) |  | Blacks | 470 | 13.5 (9.0-19.8) |  |
| **Per capita Income 2003** | 3315 |  |  | **Per capita Income 2003** | 3178 |  |  |
| under 1 MW | 1710 | 48.5 (43.7-53.3) |  | under minimum wage | 1641 | 48.6 (43.7-53.4) | 0.74 |
| above 1 MW | 1605 | 51.5 (46.7-56.3 ) |  | above minimum wage | 1537 | 51.4 (46.5-56.3) |  |
| **Per capita Income 2010** | 4176 |  |  | **Per capita Income 2010** | 4020 |  |  |
| under 1 MW | 2254 | 54.3 (51.8-56.8) |  | under minimum wage | 2158 | 54.2(51.6-56.7) | 0.18 |
| above 1 MW | 1922 | 45.7 (43.2-48.2) |  | above minimum wage | 1862 | 45.8 (43.3-48.4) |  |
|  |  |  |  |  |  |  |  |
| **Caries severity 2003** | **mean** | **(95% CI)** |  | **Caries severity 2003** | **Mean** | **(95% CI)** |  |
|  | 2.10 | (1.88-2.33) |  |  | 2.11 | (1.87-2.35) | 0.88 |
| **Caries severity 2010** | **mean** | **(95% CI)** |  | **Caries severity 2010** | **Mean** | **(95% CI)** |  |
|  | 1.72 | (1.43-2.00) |  |  | 1.70 | (1.40-2.01) | 0.78 |
| **DMFT 2003** | **mean** | **(95% CI)** |  | **DMFT 2003** | **Mean** | **(95% CI)** |  |
|  | 5.14 | (4.54-5.75) |  |  | 5.23 | (4.63-5.82) | 0.31 |
| **DMFT 2010** | **mean** | **(95% CI)** |  | **DMFT 2010** | **Mean** | **(95% CI)** |  |
|  | 3.91 | (3.41-4.40) |  |  | 3.92 | (3.41-4.44) | 0.87 |
