# Supplementary material for: Impact of water fluoridation on dental caries decline across racial and income subgroups of Brazilian adolescents
Source: Epidemiol Health. 2022 Jan 3;44:e2022007. doi: 10.4178/epih.e2022007 (PMC9016390; doi:10.4178/epih.e2022007)
Supplement: Supplementary Material 2. — Difference in Differences analysis in untreated caries presence (0 and ≥1) in Brazilian adolescents ( n=7198) [file epih-44-e2022007-suppl2.docx]

**Supplementary Materials**

| Supplementary Material 2. Difference in Differences analysis in untreated caries presence (0 and ≥1) in Brazilian adolescents ( n=7198) | | | | | | | | | | | | | | | | | |
| --- | --- | --- | --- | --- | --- | --- | --- | --- | --- | --- | --- | --- | --- | --- | --- | --- | --- |
| **Untreated Caries 0 and ≥1** | | |  |  |  |  |  |  |  |  |  |  |  |  |  |  |  |
| **Non-Fluoridation context** |  |  |  |  |  |  |  |  |  |  |  |  |  |  |  |  |  |
| **Socioeconomic groups** | **n** | **Prop with D≥1 2003** |  | **n** | **Prop with D≥1 2010** |  | **Diff-in-Diff^a^(β)** |  | **β^a^ (95% CI)** |  | **p** |  | **Diff-in-Diff^b^(β)** |  | **β^b^ (95% CI)** |  | **p** |
| under 1 MW | 937 | 74.9 |  | 1019 | 70.6 |  | -0,22 |  | −0,40/-0.03 |  | **0.002** |  | -0,3 |  | −0,49/-0.01 |  | **0.04** |
| (R) above 1 MW | 659 | 61.2 |  | 751 | 49.3 |  |  |  |  |  |  |  |  |  |  |  |  |
| **Racial groups** |  |  |  |  |  |  |  |  |  |  |  |  |  |  |  |  |  |
| Pardos+Blacks | 1174 | 71.1 |  | 1197 | 69.4 |  | (-0.77) |  | -1.25/-0.19 |  | **0.002** |  | -0.67 |  | -1.15/-0.19 |  | **0.006** |
| (R) Whites | 422 | 65.7 |  | 573 | 44.9 |  |  |  |  |  |  |  |  |  |  |  |  |
| **Fluoridation Context** |  |  |  |  |  |  |  |  |  |  |  |  |  |  |  |  |  |
| **Socioeconomic groups** |  |  |  |  |  |  |  |  |  |  |  |  |  |  |  |  |  |
| under 1 MW | 704 | 64.3 |  | 1139 | 52.0 |  | -0,18 |  | (−0,78/+0.41) |  | 0.54 |  | -0,26 |  | (-0.86/+0.33) |  | 0.39 |
| (R) above 1 MW | 878 | 54.3 |  | 1111 | 37.4 |  |  |  |  |  |  |  |  |  |  |  |  |
| **Racial groups** |  |  |  |  |  |  |  |  |  |  |  |  |  |  |  |  |  |
| Pardos+Blacks | 750 | 61.3 |  | 1170 | 52.2 |  | (-0.39) |  | (-0.97/+0.19) |  | 0.19 |  | (-0.39) |  | (-0.97/+0.18) |  | 0.18 |
| (R) Whites | 832 | 56.0 |  | 1080 | 37.2 |  |  |  |  |  |  |  |  |  |  |  |  |
| Prop - Proportion of induviduals - (R)- Reference groups | | | | |  |  |  |  |  |  |  |  |  |  |  |  |  |
| β^a^ - unadjusted β^b^ - adjusted for schooling, age, sex, eqmw and racial groups | | | | | | | | | |  |  |  |  |  |  |  |  |
