# Supplementary material for: Impact of water fluoridation on dental caries decline across racial and income subgroups of Brazilian adolescents
Source: Epidemiol Health. 2022 Jan 3;44:e2022007. doi: 10.4178/epih.e2022007 (PMC9016390; doi:10.4178/epih.e2022007)
Supplement: Supplementary Material 3. — The Difference-in-Differences analysis in average values of number of DMFT amongst Brazilian adolescents and stratified by exposure to CWF (n=7198) [file epih-44-e2022007-suppl3.docx]

**Supplementary Materials**

| Supplementary Material 3. The Difference-in-Differences analysis in average values of number of DMFT amongst Brazilian adolescents and stratified by exposure to CWF (n=7198) | | | | | | | | | | | | | | | |
| --- | --- | --- | --- | --- | --- | --- | --- | --- | --- | --- | --- | --- | --- | --- | --- |
| **DMFT Stratified by exposure to CWF** | | | | | | | | | | | | | | |  |
| **Non-Fluoridation context** | |  |  |  |  |  |  |  |  |  |  |  |  |  |  |
| **Socioeconomic groups** | **n** | **mean 2003** |  | **n** | **mean 2010** |  | **Diff-in-Diff^a^(β)** |  | **β^a^ (95% CI)** |  | **Diff-in-Diff^b^(β)** |  | **β^b^ (95% CI)** |  | **%D** |
| under 1 MW | 937 | 5.86 |  | 1019 | 5.38 |  | -0,06 |  | −0,22/+0.10 |  | -0,05 |  | -0.21; +0.11 |  | -1,02% |
| (R) above 1 MW | 659 | 5.86 |  | 751 | 5.08 |  |  |  |  |  |  |  |  |  |  |
| **Racial groups** |  |  |  |  |  |  |  |  |  |  |  |  |  |  |  |
| Browns+Blacks | 1174 | 5.78 |  | 1197 | 5.31 |  | -0.08 |  | -0.25/+0.09 |  | -0.07 |  | -0.24; +0.10 |  | -1,32% |
| (R) Whites | 422 | 6.06 |  | 573 | 5.14 |  |  |  |  |  |  |  |  |  |  |
| **Fluoridation Context** |  |  |  |  |  |  |  |  |  |  |  |  |  |  |  |
| **Socioeconomic groups** |  |  |  |  |  |  |  |  |  |  |  |  |  |  |  |
| under 1 MW | 704 | 5.10 |  | 1139 | 3.73 |  | -0,14 |  | −0,41/+0.12 |  | -0.17 |  | -0.44; +0.09 |  | -2.86% |
| (R) above 1 MW | 878 | 4.90 |  | 1111 | 3.10 |  |  |  |  |  |  |  |  |  |  |
| **Racial groups** |  |  |  |  |  |  |  |  |  |  |  |  |  |  |  |
| Browns+Blacks | 750 | 5.01 |  | 1170 | 3.74 |  | -0.18 |  | +0.44; -0.07 |  | -0.15 |  | -0.40; +0.09 |  | -3.63% |
| (R) Whites | 832 | 4.96 |  | 1080 | 3.09 |  |  |  |  |  |  |  |  |  |  |
| β - Crude coefficient; %D - Percentage of Difference ( coefficient β / baseline mean of reference (R) group) - MW – Per capita minimum-wage. DMFT – Decayed, Missing and Filled teeth; β^a^ - unadjusted β^b^ - adjusted for schooling, age, sex, income ( if analyzing racial groups) and racial groups ( if analyzing income) | | | | | | | | | | | | | | | |
