# Supplementary material for: Impact of water fluoridation on dental caries decline across racial and income subgroups of Brazilian adolescents
Source: Epidemiol Health. 2022 Jan 3;44:e2022007. doi: 10.4178/epih.e2022007 (PMC9016390; doi:10.4178/epih.e2022007)
Supplement: Supplementary Material 4. — Descriptive characteristics and weighted means of DMFT of Brazilian adolescents (n=7198) [file epih-44-e2022007-suppl4.docx]

Supplementary Material 4. Descriptive characteristics and weighted means of DMFT of Brazilian adolescents (n=7198)

| **Individual variables** | **n=7198** | **% (95%CI)** | **DMF-T** |
| --- | --- | --- | --- |
|  |  |  | **mean (95% CI)** |
| **Ethnic Group 2003 (n=3178)** |  |  |  |
| Whites | 1254 | 41.5 (36.7-46.7) | 5.17 (4.63-5.71) |
| Browns+Blacks | 1924 | 58.5 (53.6-63.3) | 5.27 (4.74-5.79) |
| **Ethnic Group 2010 (n=4020)** |  |  |  |
| Whites | 1653 | 43.0 (40.5-45.6) | 3.52 (3.19-3.85) |
| Browns+Blacks | 2367 | 57.0 (54.4-59.5) | 4.23 (3.94-4.52) |
| **Per capita Income 2003** |  |  |  |
| under minimum wage | 1641 | 48.6 (43.7-53.4) | 5.36 (4.75-5.97) |
| At and above minimum wage | 1537 | 51.4 (46.5-56.3) | 5.10(4.63-5.56) |
| **Per capita Income 2010** |  |  |  |
| under minimum wage | 2158 | 54.2(51.6-56.7) | 4.18 (3.88-4.49) |
| At and above minimum wage | 1862 | 45.8 (43.3-48.4) | 3.61 (3.30-3.93) |
| **Fluoridation 2003** |  |  |  |
| No | 1596 | 27.5 (24.5-30.6) | 5.86 (5.54-6.18) |
| Yes | 1582 | 72.5 (69.4-75.5) | 4.98 (4.47-5.50) |
| **Fluoridation 2010** |  |  |  |
| No | 1770 | 26.7 (24.8-28.8) | 5.25 (4.95-5.55) |
| Yes | 2250 | 73.3 (71.2-75.2) | 3.44 (3.16-3.71) |
| DMFT – Decayed, missing and filled Teeth |  |  |  |
