# Supplementary material for: Impact of water fluoridation on dental caries decline across racial and income subgroups of Brazilian adolescents
Source: Epidemiol Health. 2022 Jan 3;44:e2022007. doi: 10.4178/epih.e2022007 (PMC9016390; doi:10.4178/epih.e2022007)
Supplement: Supplementary Material 5. — DMFT weigthed means of Brazilian adolescents, according to Fluoridation contexts (n=7198) [file epih-44-e2022007-suppl5.docx]

| Supplementary Material 5. DMFT weigthed means of Brazilian adolescents, according to Fluoridation contexts (n=7198) | | | | |
| --- | --- | --- | --- | --- |
| **Individual variables** |  | **DMF-T** |  | **DMF-T** |
|  | **n** | **Non-fluoridated** | **n** | **fluoridated** |
| **Ethnic Group 2003** | 1596 |  | 1582 |  |
| Whites | 422 | 6.06 (5.48-6.64) | 832 | 4.96 (4.31-5.62) |
| Pardos+Blacks | 1174 | 5.78 (5.40-6.16) | 750 | 5.01 (4.23-5.78) |
| **Ethnic Group 2010** | 1770 |  | 2250 |  |
| Whites | 573 | 5.14 (4.58-5.68) | 1080 | 3.09 (2.70-3.47) |
| Pardos+Blacks | 1197 | 5.31 (4.95-5.66) | 1170 | 3.74 (3.36-4.13) |
| **Per capita Income(equivalized) 2003** | 1596 |  | 1582 |  |
| under minimum wage | 937 | 5.86 (5.47-6.24) | 704 | 5.10 (4.18-6.01) |
| above minimum wage | 659 | 5.86 (5.30-6.42) | 878 | 4.90 (4.32-5.47) |
| **Per capita Income(equivalized) 2010** | 1770 |  | 2250 |  |
| under minimum wage | 1019 | 5.38 (5.01-5.75) | 1139 | 3.73 (3.34-4.12) |
| above minimum wage | 751 | 5.08 (4.60-5.56) | 1111 | 3.10 (2.71-3.48) |
